# Ethical dilemmas in nursing professionals’ work

**DOI:** 10.1590/0034-7167.202578supl101

**Published:** 2025-09-01

**Authors:** Marcelo José dos Santos, Maristela Santini Martins, Vagner Ferreira do Nascimento

**Affiliations:** I Universidade de São Paulo, Escola de Enfermagem. São Paulo, São Paulo, Brasil; II Universidade do Estado de Mato Grosso, Faculdade Indígena Intercultural. Barra do Bugres, Mato Grosso, Brasil

Just like the nursing work process, new ways of looking at and conceiving the space of caring and being cared for have undergone important changes over the last few decades in Brazil and around the world. In particular, there has been the challenge of providing safe care in the face of constant technological advances, without at the same time demeaning: the value of touch and the principles of humanization; the epidemiological transition, with increased longevity and growth of non-degenerative chronic diseases; the ability to assist the ethnic, social and cultural diversity of Brazilian people; the judicialization of healthcare and assistance; the daily confrontation between the existential minimum and the reserve of what is possible; situations of conscientious objection at the beginning and end of life; preparation to face a pandemic; and so many other circumstances that provoked and tested nursing ´professionals’ capacity for resistance, resilience and ethics.

This work context, which often merges with nursing students’ and professionals’ life context, highlights the importance of ethical aspects in guiding individual conduct and decision-making in clinical and managerial settings. However, there are situations that are not covered by normative ethics; therefore, they are not covered solely by the code of ethics and other nursing legislation. Thus, nursing, based on theories that surround people’s health needs exclusively within their life cycles, often far from opportunities for debates about life’s vicissitudes, justice and non-technical skills, hinders professionals’ expansive, critical and reflective thinking when faced with problems, dilemmas or ethical conflicts.

Ethical problems can be conceptualized as situations that can be resolved. An ethical dilemma is based on the contradictory and mutually exclusive premise that cannot coexist, in which there will necessarily be a choice between more than one alternative. Ethical conflicts, in turn, are associated with practical situations that present incompatibility in relation to professionals’ moral values, beliefs and perceptions, which can trigger negative feelings and internal conflicts in individuals^(1)^.

In this context, powerful knowledge is required to balance these problems, dilemmas and ethical conflicts, helping nursing professionals and staff to face the problems that arise in care relationships, and here we point to bioethics as a viable and fruitful path for managing these demands. To this end, it is necessary to develop an “ethical competency”. Although there is no consensus on its definition in the literature, it can be understood in terms of strength of character and willingness to do good, attributes of those who decide to act for the benefit of others, ethical awareness associated with sensitivity, to identify an ethical issue, and ability to make moral judgment, an attribute of those who are autonomous in the way they think and act^(2)^.

Ethical competency is constituted by the attributes of ethical knowledge, understood as the integration of philosophical and practical knowledge, considering the contexts and individuals involved, and by ethical sensitivity, which enables the recognition of a possible question or problem, conflict and ethical dilemma in situations. These attributes support an ethical reflection, which requires the consideration of several alternatives, enabling ethical decision-making, which results in a reasonable and responsible choice between available alternatives, i.e., ethical actions and behaviors, motivated by the good and respect for others^(3)^.

This competency must also be developed during the training of nursing professionals. To this end, it is essential to have qualified trainers, since simply existing is no guarantee that professionals will know and apply ethical knowledge, and an example of this is the constant complaints and notifications to regulatory bodies, as well as so many other cases that still remain “invisible” to intervention, but generate suffering, violence and even death (in various forms).

## References

[1] Beauchamp TL, Childress JF. (2011). Princípios de ética biomédica.

[2] Kulju K, Stolt M, Suhonen R, Leino-Kilpi H. (2016). Ethical competence: a concept analysis. Nursing Ethics.

[3] Lechasseur K, Caux C, Dollé S, Legault A. (2018). Ethical competence: an integrative review. Nursing Ethics.

