# Supplementary material for: Ethical dilemmas in nursing professionals’ work
Source: Rev Bras Enferm. 2025 Sep 1;78(Suppl 1):e78supl101. doi: 10.1590/0034-7167.202578supl101 (PMC12407885; doi:10.1590/0034-7167.202578supl101)
Supplement: Translation

# Translation: Dilemas éticos no trabalho dos profissionais de enfermagem

**DOI:** 10.1590/0034-7167.202578supl101pt

**Authors:** Marcelo José dos Santos, Maristela Santini Martins, Vagner Ferreira do Nascimento

**Affiliations:** I Universidade de São Paulo, Escola de Enfermagem. São Paulo, São Paulo, Brasil; II Universidade do Estado de Mato Grosso, Faculdade Indígena Intercultural. Barra do Bugres, Mato Grosso, Brasil

Assim como o processo de trabalho de enfermagem, novas formas de olhar e conceber o espaço do cuidar e do ser cuidado sofreram importantes mudanças ao longo das últimas décadas no Brasil e no mundo. Especialmente, houve o desafio de realizar cuidados seguros diante de constantes avanços tecnológicos, sem ao mesmo tempo desmerecer: o valor do toque e dos princípios da humanização; a transição epidemiológica, com aumento da longevidade e crescimento de doenças crônicas não degenerativas; a competência em assistir a diversidade étnica, social e cultural do povo brasileiro; a judicialização de cuidados e assistência à saúde; o confronto diário entre o mínimo existencial e a reserva do possível; as situações de objeção de consciência no início e final de vida; o preparo para enfrentar uma pandemia; e tantas outras circunstâncias que provocaram e testaram a capacidade de resistência, resiliência e ética dos profissionais de enfermagem.

Esse contexto de trabalho, que muitas vezes mescla com o contexto de vida do estudante e do profissional de enfermagem, exalta a importância dos aspectos éticos no balizamento de condutas individuais e tomadas de decisão no âmbito clínico e gerencial. No entanto, há situações que não estão previstas na ética normativa; logo, não são contempladas somente no código de ética e demais legislações de enfermagem. Assim, o fazer enfermagem, fundamentado em teorias que cercam as necessidades de saúde das pessoas exclusivamente dentro dos seus ciclos de vida, frequentemente longe de oportunidades de debates sobre vicissitudes da vida, justiça e competências não técnicas, dificulta o pensamento expansivo, crítico e reflexivo do profissional diante de problemas, dilemas ou conflitos éticos.

Os problemas éticos podem ser conceituados como situações possíveis de resolução. Já o dilema ético parte da premissa contraditória e mutuamente excludente que não pode coexistir, em que necessariamente haverá a escolha entre mais de uma alternativa. Os conflitos éticos, por sua vez, estão associados a situações da prática que apresentem incompatibilidade em relação aos valores morais, crenças e percepções do profissional, que podem desencadear sentimentos negativos e conflitos internos no indivíduo^(1)^.

Nesse âmbito, exige-se um saber potente na equalização desses problemas, dilemas e conflitos éticos, auxiliando os profissionais de enfermagem e equipe no enfrentamento de problemáticas que suscitam nas relações de cuidado, e aqui apontamos a bioética como um caminho viável e frutífero para o gerenciamento dessas demandas. Para tanto, é necessário desenvolver uma “competência ética”. Embora não exista um consenso sobre sua definição na literatura, esta pode ser compreendida em termos de força de caráter e disposição de fazer o bem, atributos de quem decide agir em benefício de outras pessoas, consciência ética associada à sensibilidade, para identificar uma questão ética, e habilidade de julgamento moral, atributo de quem é autônomo na forma de pensar e agir^(2)^.

A competência ética é constituída pelos atributos do conhecimento ético, entendida como a integração de saberes filosóficos e práticos, considerando os contextos e indivíduos envolvidos, e pela sensibilidade ética, que possibilita o reconhecimento de uma possível questão ou problema, conflito e dilema ético nas situações. Esses atributos sustentam uma reflexão ética, que exige a consideração de várias alternativas, possibilitando a tomada de decisão ética, que resulta em uma escolha razoável e responsável entre as alternativas disponíveis, ou seja, ações e comportamentos éticos, motivados para o bem e para o respeito aos outros^(3)^.

É necessário que essa competência também seja desenvolvida durante a formação dos profissionais de enfermagem. Para isso, é imprescindível que haja formadores capacitados, já que simplesmente existir não é uma garantia que os profissionais conheçam e apliquem os saberes éticos, e um exemplo disso está nas constantes denúncias e notificações junto a órgãos fiscalizadores, bem como tantos outros casos que ainda seguem “invisíveis” à intervenção, mas gerando sofrimentos, violências e inclusive mortes (de diversas formas).

# Translation: Dilemas éticos en el trabajo de los profesionales de enfermería

**DOI:** 10.1590/0034-7167.202578supl101es

**Authors:** Marcelo José dos Santos, Maristela Santini Martins, Vagner Ferreira do Nascimento

**Affiliations:** I Universidade de São Paulo, Escola de Enfermagem. São Paulo, São Paulo, Brasil; II Universidade do Estado de Mato Grosso, Faculdade Indígena Intercultural. Barra do Bugres, Mato Grosso, Brasil

Al igual que el proceso de trabajo de la enfermería, nuevas formas de mirar y concebir el espacio de cuidar y ser cuidado sufrieron importantes cambios en las últimas décadas en Brasil y en el mundo. En particular, estaba el desafío de brindar una atención segura frente a los constantes avances tecnológicos, sin menoscabar al mismo tiempo: el valor del tacto y los principios de humanización; la transición epidemiológica, con aumento de la longevidad y crecimiento de enfermedades crónicas no degenerativas; la competencia para atender la diversidad étnica, social y cultural del pueblo brasileño; la judicialización de la atención y asistencia sanitaria; el enfrentamiento cotidiano entre el mínimo existencial y la reserva de lo posible; las situaciones de objeción de conciencia al inicio y al final de la vida; la preparación para enfrentar una pandemia; y las muchas otras circunstancias que provocaron y pusieron a prueba la resistencia, la resiliencia y la ética de los profesionales de enfermería.

Este contexto de trabajo, que muchas veces se fusiona con el contexto de vida del estudiante y del profesional de enfermería, resalta la importancia de los aspectos éticos en la orientación de la conducta individual y la toma de decisiones en el ámbito clínico y gerencial. Sin embargo, hay situaciones que no están cubiertas por la ética normativa; por lo tanto, no sólo están cubiertos por el código de ética y otras leyes de enfermería. Así, la práctica de enfermería, basada en teorías que rodean las necesidades de salud de las personas exclusivamente dentro de sus ciclos vitales, muchas veces lejos de oportunidades para debates sobre las vicisitudes de la vida, la justicia y las habilidades no técnicas, dificulta que los profesionales piensen de manera expansiva, crítica y reflexiva frente a problemas, dilemas o conflictos éticos.

Los problemas éticos pueden conceptualizarse como situaciones que pueden resolverse. El dilema ético se basa en la premisa contradictoria y mutuamente excluyente de que no pueden coexistir, en la que necesariamente habrá que elegir entre más de una alternativa. Los conflictos éticos, a su vez, están asociados a situaciones prácticas que presentan incompatibilidad con los valores, creencias y percepciones morales del profesional, lo que puede desencadenar sentimientos negativos y conflictos internos en los individuos^(1)^.

En este contexto, se requiere conocimiento potente para equiparar estos problemas, dilemas y conflictos éticos, ayudando a los profesionales y personal de enfermería a enfrentar los problemas que surgen en las relaciones de cuidado, y aquí apuntamos a la bioética como un camino viable y fructífero para la gestión de estas demandas. Para ello es necesario desarrollar la “competencia ética”. Aunque no existe consenso sobre su definición en la literatura, puede entenderse en términos de fortaleza de carácter y voluntad de hacer el bien, atributos de quien decide actuar en beneficio de otras personas, conciencia ética asociada a la sensibilidad, para identificar una cuestión ética, y capacidad de emitir juicio moral, atributo de quien es autónomo en su forma de pensar y actuar^(2)^.

La competencia ética está constituida por los atributos del conocimiento ético, entendido como la integración del conocimiento filosófico y práctico, considerando los contextos e individuos involucrados, y por la sensibilidad ética, que posibilita el reconocimiento de una posible cuestión o problema, conflicto y dilema ético en situaciones. Estos atributos sustentan la reflexión ética, que requiere la consideración de varias alternativas, posibilitando la toma de decisiones éticas, que resultan en una elección razonable y responsable entre las alternativas disponibles, es decir, acciones y comportamientos éticos, motivados por el bien y el respeto por los demás^(3)^.

Esta competencia también debe desarrollarse durante la formación de los profesionales de enfermería. Para ello es fundamental que existan formadores capacitados, ya que el solo existir no es garantía de que los profesionales conozcan y apliquen conocimientos éticos, y un ejemplo de ello está en las constantes denuncias y notificaciones a órganos de control, así como muchos otros casos que aún permanecen “invisibles” a la intervención, pero generando sufrimiento, violencia e incluso muertes (de diferentes maneras).
